# Profiling insecticide resistance phenotypes and genotypes in *Aedes aegypti* populations across four regions in Puerto Rico

**DOI:** 10.1038/s41598-025-03709-x

**Published:** 2025-07-18

**Authors:** Emma L. Collins, Joanelis Medina Quintana, Reynaldo Morales, Sophie Moss, Holly Acford-Palmer, Matthew Higgins, Jody Phelan, Taane G. Clark, Grayson Brown, Susana Campino

**Affiliations:** 1 Faculty of Infectious and Tropical Diseases, London School of Hygiene & Tropical Medicine, Keppel Street, London, UK; 2 Puerto Rico Vector Control Unit, Puerto Rico, USA; 3 Faculty of Epidemiology and Population Health, London School of Hygiene & Tropical Medicine, London, UK

**Keywords:** Insecticide resistance, Arbovirus vector control, Molecular surveillance, *Aedes aegypti*, Genetic markers, DNA sequencing

## Abstract

Vector-borne diseases exert a considerable toll on global health. The efficacy of vector control strategies is being threatened by the emergence and spread of insecticide resistance worldwide. In this study, we collected *Aedes aegypti* mosquitoes from five regions of Puerto Rico to investigate their insecticide resistance phenotypes and genotypes.  Insecticide resistance intensity CDC bioassays were employed to determine the response to deltamethrin and malathion. In parallel, next generation targeted amplicon sequencing was used to investigate the presence of insecticide resistance-conferring mutations in nine targets across four genes: the voltage gated sodium channel (*vgsc*); GABA receptor (resistance to dieldrin, rdl); acetylcholinesterase (*ace-1*); and glutathione-S-transferase epsilon 2 (*GSTe2*). We observed high resistance intensity to deltamethrin and malathion in *Ae. aegypti* mosquitoes Resistance was supported by molecular evidence revealing five mutations (V410L (*vgsc*), V1016I/G (*vgsc*), F1534C (*vgsc*), A296S (*rdl*)), previously linked to insecticide resistance. A previously undocumented mutation, L944I (L921I in *Ae. aegypti*, *vgsc*), was identified. While not yet reported in *Aedes* spp. vectors, this mutation has been associated with pyrethroid resistance in other medically important vectors and agricultural pests. Our research highlights the presence of insecticide resistance and associated mutations in Puerto Rico, which is valuable for vector control programs, providing information to guide decisions regarding the implementation of effective control interventions.

**Supplementary Information:**

The online version contains supplementary material available at 10.1038/s41598-025-03709-x.

## Introduction

Vector-borne diseases (VBDs) cause vast morbidity and at least 700,000 deaths annually worldwide^1^. The majority of VBDs are transmitted by mosquitoes from three genera (*Anopheles*,* Culex* and *Aedes)*. Following *Anopheles* mosquitoes, which are primary vectors for malaria parasites, *Aedes* mosquitoes, notably *Ae. aegypti* (L.), stand as a significant contributor to the global disease burden. *Ae. aegypti* is the dominant vector of many arboviruses including Zika, dengue, yellow fever, and Chikungunya viruses. Millions of cases of arboviral diseases occur annually, nearly 400 million from dengue alone^2^. These diseases impose significant social and economic burden across the tropics, with the Americas being particularly affected with 3,126,573 cases reported in 2022^3^. In Puerto Rico, arboviruses such as dengue and Zika have been responsible for substantial outbreaks^4,5^. Chikungunya and Zika were introduced to the island in 2014 and 2015, respectively^4^, while dengue maintains endemic status, with an annual average of 5,000 to 7,000 cases^6^. Despite this baseline prevalence, dengue outbreaks occur regularly, notably in 2007, 2010 and 2013 where approximately 20,000 cases were reported in each year^5^.

Insecticides have been used to effectively control vector populations and reduce the associated disease burden, particularly through the use of insecticide treated nets to combat malaria. There are currently nine classes of insecticide used globally against mosquitoes, including pyrethroids, carbamates, organophosphates, organochlorines, neonicotinoids, pyrroles, butenolides, juvenile hormone mimics and spinosyns^7,8^. Unfortunately, the use of insecticides for both vector control and agriculture has led to the rise of insecticide resistance globally, threatening control programs. In Puerto Rico, vector control measures are applied inconsistently, targeting both adult mosquitoes and larvae. Pyrethroid resistance in *Ae. aegypti* has already been documented on the island^9–11^, and mutations linked to this resistance—such as V1016I and F1534C in the voltage-gated sodium channel (*vgsc*) gene—have been detected at high frequencies^11^. Additionally, evidence of metabolic resistance mechanisms has been found using synergist assays to isolate the action of detoxifying enzymes, in this case piperonyl butoxide, as well as the use of RNA sequencing (RNA-seq) to identify the upregulation of cytochrome P450 genes^9,12^.

Assessing phenotypic resistance is essential to inform vector control programmes and support the implementation of the most effective methods. Conducting bioassays, including well-established methods like the WHO tube tests^13^ and WHO or CDC bottle bioassays^13,14^ to evaluate mosquito mortality following insecticide exposure, can be a time-intensive process, the judgement of knockdown can be difficult, and comparisons across diverse studies or between WHO and CDC methodologies can be challenging^7^. Consequently, there is growing emphasis on monitoring molecular markers of resistance, as advocated by the WHO, highlighting the importance of understanding molecular mechanisms for designing effective vector control strategies^7^. These methodologies can act as an early warning system to show emergence of resistance before control methodologies lose complete efficacy.

Molecular methodologies have become a cost-effective approach for the monitoring of insecticide resistance, particularly when using multiplex assays that target many loci in parallel. Targeted amplicon next-generation sequencing (Amp-seq) offers the possibility to analyse a large number of candidate genetic regions across many samples using next-generation sequencing platforms^15–18^. It offers increased sensitivity compared to PCR-RFLP and real-time PCR, which in general only target a few markers, and decreased costs in comparison to whole genome sequencing (WGS). This approach will not only offer insights into the status of insecticide resistance but also aid in the identification of new resistance markers.

In this study we use bioassays to investigate the response of *Ae. aegypti* populations in Puerto Rico to deltamethrin, which targets the voltage gated sodium channels, and malathion, which inhibits acetylcholinesterase when activated to malaoxon^19^. We complement the study with molecular surveillance using a multi target Amp-seq approach, previously validated by us, to identify molecular markers associated with insecticide resistance in *Ae. aegypti* collected across five regions in Puerto Rico^16^. We targeted regions in *ace-1*,* vgsc*,* rdl* and *GSTe2* genes, with mutations associated with insecticide resistance. We used dual-indexing barcodes to allow for high-throughput processing and enable sequencing of multiple samples simultaneously to decrease costs.

## Results

### Mosquito collection and insecticide resistance assessment

**Fig. 1:**
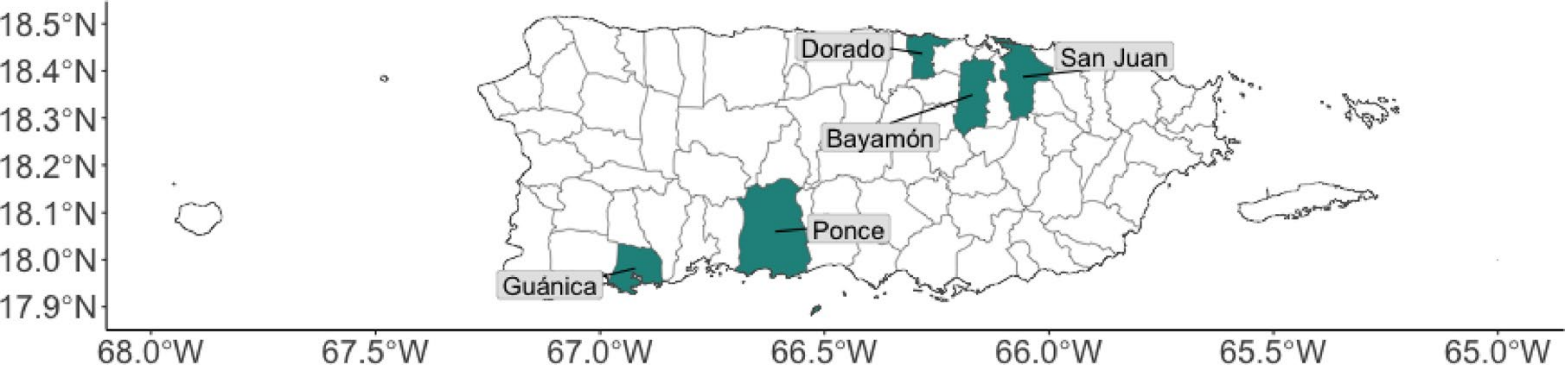
Sampling locations for ovitraps.

Over 5,000 eggs were collected from Bayamon, Dorado, Guánica, Ponce and San Juan between 5th April 2022 and 1st June 2022 (Fig. 1) and reared to adults. As insufficient eggs were collected from Guánica, bioassays were not performed for mosquitoes in this region. The mosquito populations in the other 4 regions were tested for susceptibility to deltamethrin and malathion using CDC bottle bioassays with a diagnostic dose and time of 0.75 µg/bottle, 30 min and 400 µg/bottle, and 15 min, respectively as per CDC recommendations (Table 1)^20^. A total of 1,003 mosquitoes (765 field mosquitoes) were exposed to deltamethrin and a further 925 (686 field mosquitoes) were exposed to malathion, this includes control strain and unexposed field caught controls.

**Table 1:** Percentage mortality and number of mosquitoes (n) included in each bioassay with differing times the diagnostic dose to deltamethrin and malathion in mosquitoes collected in 2022 across 4 regions. Diagnostic dose and times for deltamethrin were 0.75 µg/ml and 30 min, while it was 400 µg/ml and 15 min for malathion. ROCK is the susceptible colony control.

Deltamethrin Mortality % (*n*)					
Location	Wild No exposure	ROCK (0.75 µg/bottle)	x1 (0.75 µg/bottle)	x5 (3.75 µg/bottle)	x10 (7.5 µg/bottle)
Bayamon (*n* = 211)	0.0 (91)	97.4 (78)	22.7 (97)	42.2 (64)	90 (50)
Dorado (*n* = 129)	0.0 (36)	100 (45)	2.2 (45)	46.7 (45)	87.2 (39)
Ponce (*n* = 136)	0.0 (46)	97.5 (40)	40.4 (47)	76.2 (42)	93.6 (47)
San Juan (*n* = 289)	1.1 (88)	100 (73)	21.4 (98)	41.8 (91)	89 (100)
Total	0.3 (261)	98.7 (236)	22.0 (287)	48.8 (242)	89.8 (236)

Malathion Mortality % (n)					
Location	Wild No exposure	ROCK (400 µg/bottle)	x1 (400 µg/bottle)	x2 (800 µg/bottle)	x3 (1200 µg/bottle)
Bayamon (*n* = 164)	0.0 (70)	96.1 (51)	36.4 (66)	51 (49)	83.7 (49)
Dorado (*n* = 183)	0.0 (105)	100 (45)	17.6 (91)	50.7 (69)	82.6 (23)
Ponce (*n* = 122)	0.0 (48)	95.2 (42)	87.2 (47)	91.7 (48)	100 (27)
San Juan (*n* = 217)	0.0 (60)	72.4* (69)	40 (70)	61.4 (70)	62.3 (77)
Total	0.0 (283)	88.9 (207)	39.8 (274)	62.3 (236)	76.7 (176)

**Fig. 2:**
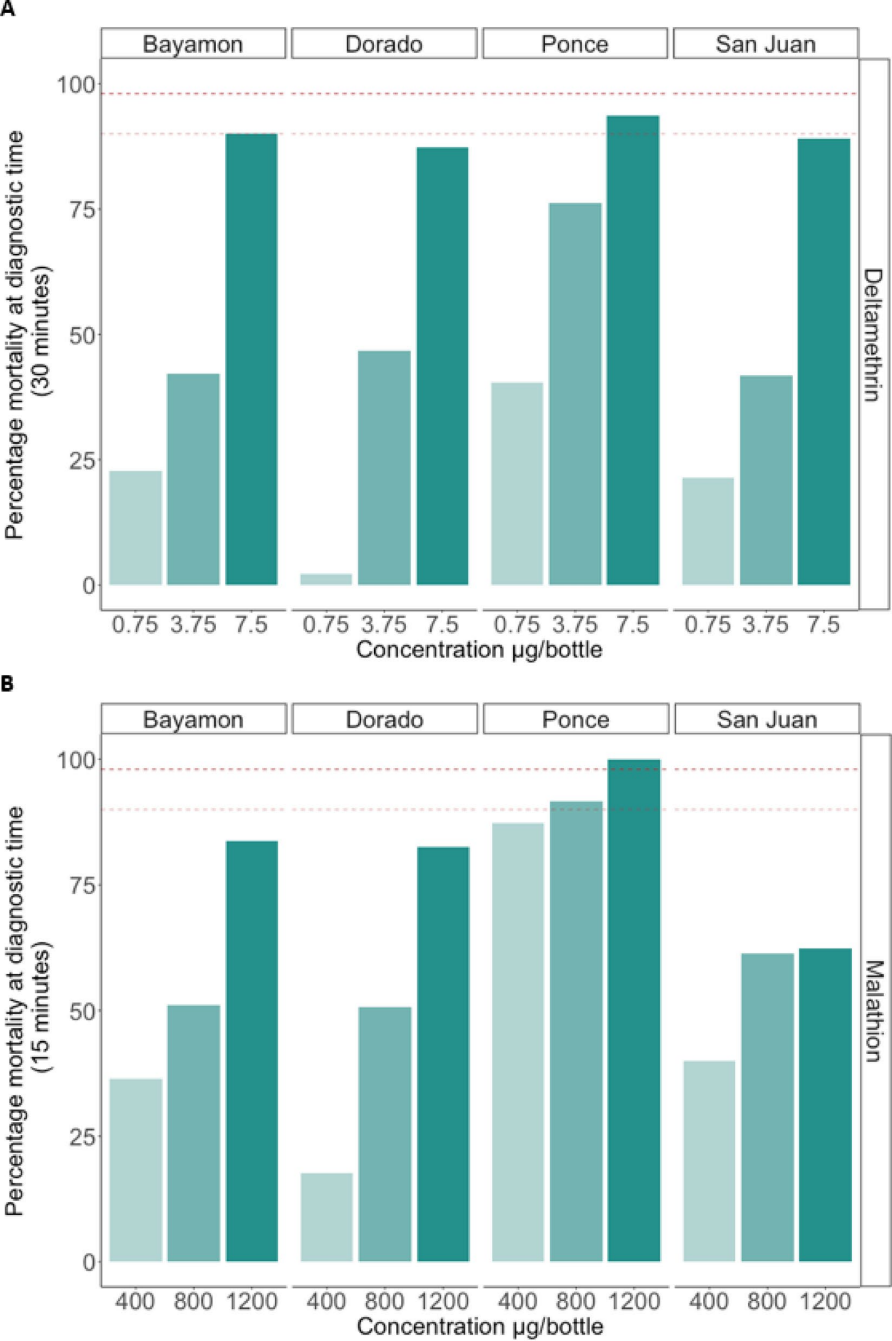
Mortality for the CDC bioassays at each concentration to (**A**) deltamethrin and (**B**) malathion for each location. Dotted red line shows 98% mortality above which indicates susceptibility and light red dotted line shows 90% mortality, between 90 and 98% indicates possible resistance.

The implementation of insecticide bioassays to deltamethrin displayed high intensity of resistance (Fig. 2A). The lowest rate of mortality was observed in Dorado with only 2.2% mortality against the diagnostic dose, followed by San Juan and Bayamon with 21.4% and 22.7% mortality, respectively. Finally, the *Ae. aegypti* population in Ponce showed the highest mortality rate of 40.4%, however, this rate falls well below the 98% mortality threshold commonly considered indicative of susceptibility^21^. Mortality in all four regions increased when mosquitoes were subjected to five times the diagnostic dose of insecticide. However, these rates remain far below susceptible levels of mortality.

Exposure to malathion showed higher mortality rates than exposure to deltamethrin, however, resistance was still observed (Fig. 2B). Like the results for deltamethrin, the Ponce population showed the highest mortality rate against the diagnostic dose of malathion (87.2%), followed by San Juan (40.0%), Bayamon (36.4%), and Dorado (17.6%). Every location’s mortality rate increased with exposure to a higher dose of insecticide. The only population to reach the 98% mortality threshold was the population from Ponce after exposure to three times the diagnostic dose (1200 µg/ml). As the number of mosquitos varied between sites, a multivariate generalised log-linear model (Poisson regression) was fitted with (log) mortality count as the outcome, the (log) number of mosquitoes as an offset, with location, concentration and insecticide included as covariates. This approach indicated that, as expected, concentration (*p* = 2 × 10^− 16^) and insecticide (*p* = 1.46 × 10^− 7^) affect the rate of mortality, with malathion having a greater effect than deltamethrin (coefficient estimate = 0.430, deltamethrin as reference). It revealed that the mortality rate in Ponce was higher than the reference location Bayamon (coefficient estimate = 0.343, *p* = 1.8 × 10^− 5^), while comparisons for San Juan and Dorado showed no differences (*p* > 0.07).

### Amplicon sequencing to identify molecular markers associated with resistance

A total of 178 samples were sequenced across 10 amplicons, covering *cytochrome oxidase* (COI) for speciation, and 4 genes (*vgsc*,* rdl*,* ace-1* and *GSTe2*) associated with insecticide resistance. The average sequencing coverage observed across the amplicons was 190.5-fold but varied across the ten amplicons (range: 6.5 to 550.8-fold). The lowest coverage was observed in the longest amplicon which targets *cytochrome oxidase* gene for mosquito speciation (LCO1490 and HCO1298)^22^ (Table S3). The average amplicon length was 453 bp (range: 321 to 709 bp). All 178 samples passed quality control filters, and included 51 from Bayamon, 42 from Dorado, 7 from Guánica, 33 from Ponce, and 45 from San Juan.

Amongst the 178 samples screened, 57 SNPs were identified, of which 14 were non-synonymous, one was in a splice region and 14 were synonymous, while the remainder were detected in intronic regions (Table 2, Table S4). Previously identified insecticide resistance SNPs will be referenced according to the organism in which they were first reported. However, *Ae. aegypti*-specific nomenclature is provided in Table S5 for ease of reference. The non-synonymous mutations (*n* = 14) were identified across three genes (*ace-1*,* vgsc*, and *rdl)*, the majority of these occurred in the *vgsc* gene (*n* = 12), while there was a single non-synonymous in both the *ace-1* (*n* = 1) and *rdl* (*n* = 1) genes (Table 2). No non-synonymous mutations were identified in *GSTe2.* Most of these synonymous mutations (*n* = 14) were found in the *vgsc* gene (*n* = 9) while the remainder were in the *ace-1* gene (*n* = 5) (Table S4). The synonymous 506T mutation detected in the *ace-1* gene has previously been documented in resistant *Ae. aegypti* mosquitoes in Indonesia (not in combination with G119S)^23^, however functional work has not confirmed its association with resistance.

**Table 2:** Summary of the non-synonymous mutations identified in the 178 samples screened. Nomenclature as per *Ae. Aegypti. Rdl* mutations were based on the AAEL008354-RF transcript, *Vgsc* mutations utilised the AAEL023266-RLtranscript and *ace-1* mutations refer to AAEL034366-RD transcript. This ^ symbol indicates a previously described mutation associated with insecticide resistance. A * indicates a deletion.

Chrom	Gene	Position	Nucleic acid change	Annotation	Codon position in ref.	Genotype (*n*)			Alternative allele frequency (%)
						Homo. ref	Hetero.	Homo. alt	
2	*rdl*	41,847,790	G > T	A296S^	301	7	24	28	67.7
3	*ace-1*	161,500,150	C > T	A482T	482	105	1	0	0.47
3	*vgsc*	315,931,756	A > C	I1845S	1854	115	1	0	0.43
3	*vgsc*	315,931,943	G > A	Q1805*	1814	73	6	0	3.61
3	*vgsc*		G > C	Q1805E			2	0	1.20
3	*vgsc*		G > T	Q1805K			2	0	1.20
3	*vgsc*	315,932,144	C > T	G1738S	1747	89	1	0	0.56
3	*vgsc*	315,932,210	C > A	V1716L	1725	88	2	0	1.11
3	*vgsc*	315,939,224	A > C	F1554C^	1534	0	18	109	92.91
3	*vgsc*	315,983,762	A > C	**	1016	50	5	0	4.55
3	*vgsc*	315,983,763	G > T	**	1016	0	5	50	95.45
3	*vgsc*	315,984,130	A > C	F967C	979	50	8	0	6.90
3	*vgsc*	315,998,386	A > T	F943Y	932	137	1	0	0.36
3	*vgsc*	315,998,453	A > T	L921I^	910	95	30	2	22.08
3	*vgsc*	315,998,530	A > C	L895R	1008	118	6	0	2.42
3	*vgsc*	316,080,722	C > A	V408L^	410	0	15	101	93.53

**In 48 samples there was no mutation at position 315,983,762 however there was a mutation at 315,983,673 which resulted in a V1016I (ATA). While 5 samples had both a mutation at 315,983,762 and 315,983,763 which resulted in a heterozygous V1016G/V1016I genotype (GGA/ATA). In 2 samples for each mutation position, there was only at that position and not the other so we cannot say what the resulting amino acid would be in that codon.

Reference: GTA/CAT -> V1016.

315,983,762: A > C, GGA/CCT -> Glycine V1016G.

315,983,763: G > T, ATA/TAT -> Isoleucine V1016I.

Five non-synonymous mutations were detected have previously been associated with insecticide resistance (*rdl* A296S; *vgsc* F1534C, V1016I, V1016G, V410L) (Table 2). The *rdl* A296S mutation was found in 52 samples, of which 24 had heterozygous genotypes. This mutation has been found in multiple insects including *Drosophila*, *Anopheles* and *Aedes* species, and is associated with resistance to dieldrin^16,24–27^. The four *vgsc* mutations observed are associated with resistance to the pyrethroid insecticide class. The *vgsc* F1534C mutation was present in 127 samples of which 18 had heterozygous genotypes (*n* = 127). The V1016I *vgsc* mutation presented as homozygous alternative in 48 samples (*n* = 53). The V1016G mutation was less frequent, with only five samples having this mutation in one chromosome, alongside the V1016I mutation in the other chromosome (V1016G/I). One hundred and one samples were homozygous alternative for the *vgsc* V410L, as well as 15 with heterozygous genotypes (*n* = 116).

The *vgsc* L944I (L921I *Ae. aegypti* AAEL023266-RL transcript numbering) mutation was identified in our samples. This mutation is of particular interest because, although it has not been previously reported in *Ae. aegypti*, the equivalent amino acid change (L925I) has been documented in several other arthropod species, including *Triatoma infestans* and *Bemisia tabaci*^28,29^. The mutation allele frequency was 22.1% with 30 heterozygotes identified and 2 homozygous alternatives (*n* = 127).

**Fig. 3:**
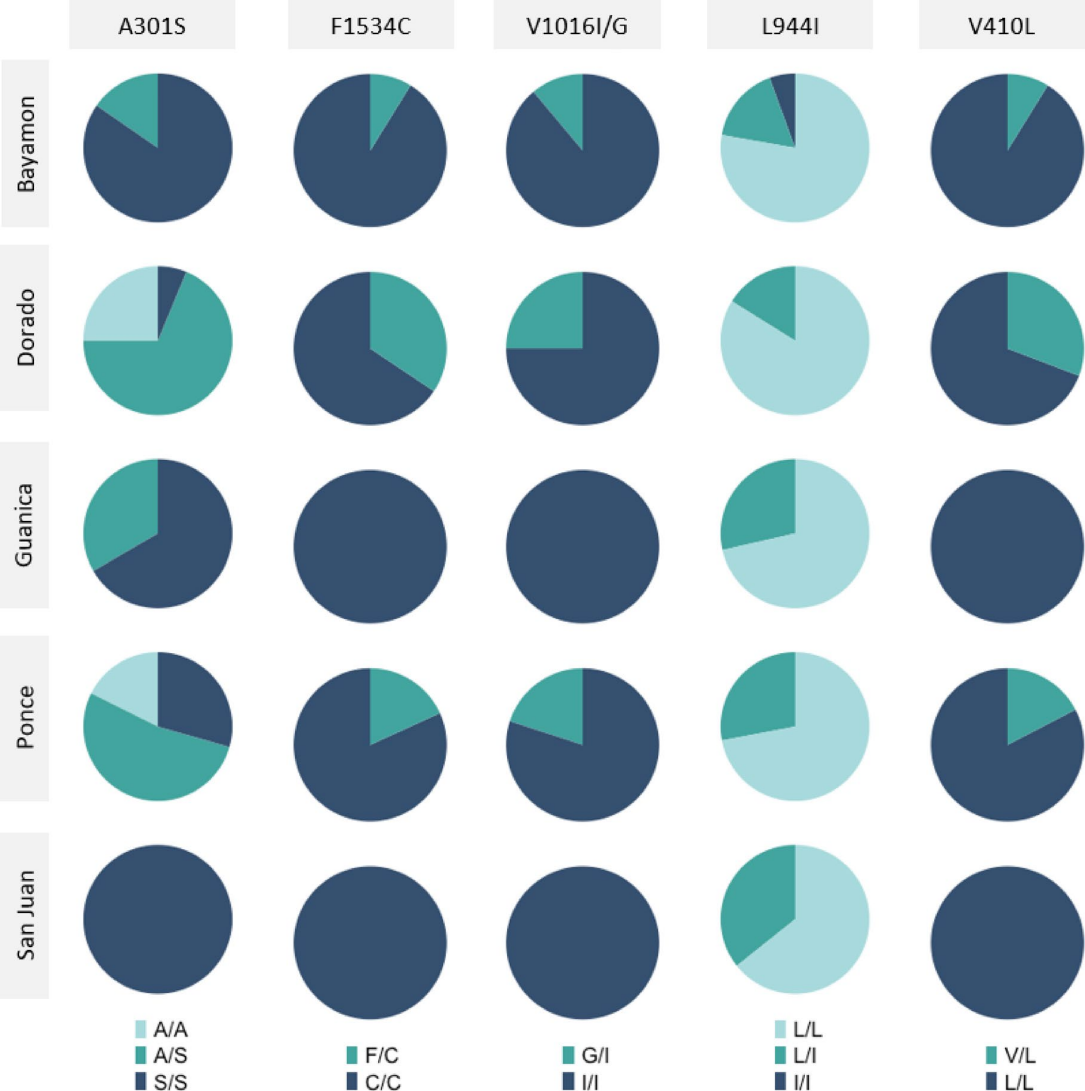
The amino-acid proportions for the five main insecticide resistance-associated mutations across all locations. For the V1016 codon, a combination of the SNPs at positions 315983762 and 315983763 was used to identify the consequence.

Five of the non-synonymous SNPs associated with insecticide resistance were detected in the 5 locations (Fig. 3). V1016G was the only mutation not detected in all locations, being absent from Guánica and San Juan. The *vgsc* V410L mutation appears fixed in both San Juan and Guánica and is approaching fixation in Bayamon and Ponce (Table 3). Dorado had the lowest proportion of homozygous alternative mutations for *vgsc* V410L, though the allele was still observed at a frequency of 84.6%. The F1534C and V1016I had the next highest overall allele frequencies of 92.9% and 95.5%, respectively. Overall, there were minimal differences in allele frequencies between the insecticide resistance SNPs and the sampling locations (χ² test, *p* > 0.05), suggesting that the observed phenotypic differences may not be primarily driven by the detected SNPs. Spearman’s-rank analysis between mortality rate and allele frequency for each insecticide resistance SNP identified by location indicated no correlation (*p* > 0.30).

**Table 3:** Allele frequency (%) of each of the six detected insecticide resistance-associated mutations in each of the locations.

Gene	Mutation (position)	Overall	Bayamon	Dorado	Guánica	Ponce	San Juan
*rdl*	A296S (41847790)	66.8	92.3	40.6	83.3	55.9	100
*vgsc*	F1534C (315939224)	92.9	95.6	82.8	100	90.9	100
*vgsc*	V1016G (315983762)	4.7	5.6	11.1	0.0	10.0	0.0
*vgsc*	V1016I (315983763)	95.3	94.4	88.9	100	90.0	100
*vgsc*	L944I (315998453)	13.4	13.9	8.1	14.3	14.0	17.9
*vgsc*	V410L (316080722)	93.5	95.5	84.6	100	91.3	100

### Linked mutations

**Fig. 4:**
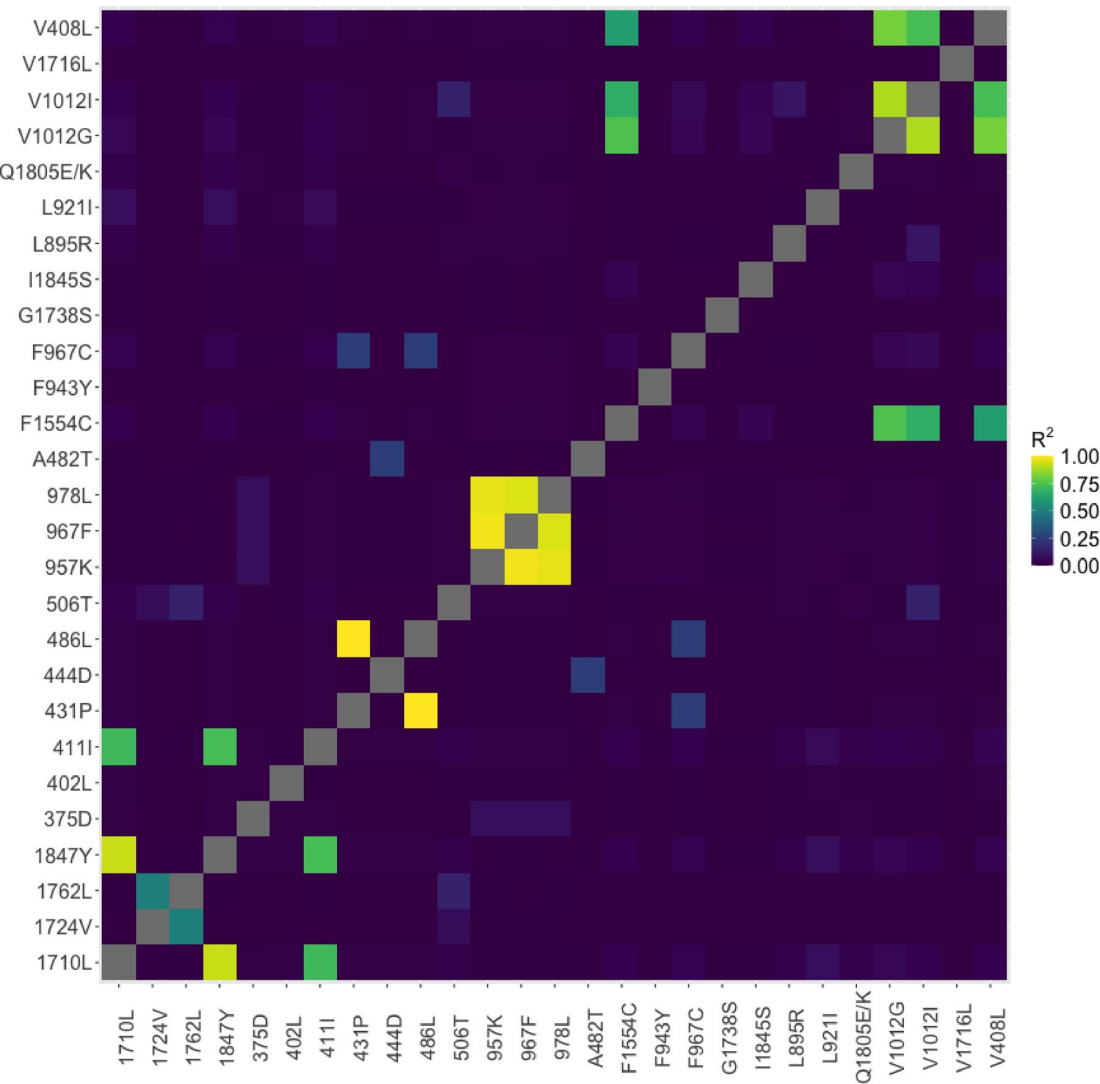
Linkage disequilibrium R^2^ values between synonymous and non-synonymous positions.

Linkage disequilibrium analysis was only carried out on chromosome 3 due too few SNPs being identified on chromosome 2. Overall, 236 pairwise SNP combinations of the 3,136 possible, have an R^2^ of more than 0.8^30^, which includes 33 unique SNP locations (Table S6). Figure 4 shows the R^2^ values for synonymous and non-synonymous SNPs. Most of these SNPs were in the *vgsc* gene (*n* = 34), while two were identified in *ace-1.* Strong linkage was identified between four non-synonymous mutations located in *vgsc* (V410L, V1016I, V1016G, and F1534C) (R^2^ range: 0.61–0.90).

## Discussion

This study provides strong evidence of both phenotypic and genotypic insecticide resistance in Puerto Rico, using a combination of bioassays and a targeted Amp-seq assay. Bioassays were conducted on *Ae. aegypti* samples from four regions, and Amp-seq assays on samples from five regions across Puerto Rico. We observed elevated levels of phenotypic resistance to both deltamethrin and malathion, along with the detection of five genetic markers linked to resistance against organochlorines (cyclodienes and phenylpyrazoles) and pyrethroids.

A 2016 study in Puerto Rico identified resistance in *Aedes aegypti* populations to permethrin, phenothrin, etofenprox, and tetramethrin through phenotypic bioassays^10^. Hemme et al. (2019) reported widespread resistance, with deltamethrin achieving effective control in only five out of 38 regions, and no populations showing susceptibility to malathion^10^. Naled was the most effective insecticide, killing 100% of mosquitoes in all locations tested. This study updates those findings and further examines the intensity of resistance to deltamethrin and malathion. Significant resistance was observed at up to three times and ten times the diagnostic doses for malathion (1200 µg/bottle) and deltamethrin (7.5 µg/bottle), respectively.

Pyrethroid-associated mutations have also been described previously (V1016I and F1534C) at high frequency in Puerto Rico (60-100% and 80-100% respectively)^11^. This study supports previous findings by identifying the V1016I and F1534C SNPs, with allele frequencies ranging from 87.5 to 100% and 82.8–100%, respectively, similar to those reported by Ponce-García et al. (2016). Multiple mutations have been described in the V1016 codon, including a substitution at position 315,983,762 resulting in V1016G, and another at position 315,983,763, leading to V1016I. The V1016I mutation has primarily been detected in Africa and the Americas^31,32^, while V1016G is more prevalent in Asia^33,34^. In our dataset, V1016I was the most common genotype, detected at a frequency of 95.5%. However, we also identified a combination of mutations at both positions (315983762 and 315983763), though these mutations occurred on separate chromosomes. This compound heterozygous arrangement was found at low frequency (4.5%, *n* = 5) with the resulting genotype V1016G/V1016I. The presence of multiple mutations at this codon suggest it may be under selective pressure^35^. The detection of the V1016G mutation is particularly notable, given its historical restriction to Asia^36^. More recently, however, V1016G has also been reported in Benin, Panama and USA ^37–39^, suggesting a potential breakdown in the geographical segregation of these SNPs.

This study identified additional insecticide resistance mutations in the *vgsc* gene within this population that had not previously been reported in Puerto Rico, including the V410L, 1016G mutations and a novel L944I mutation. However, it may be noted that the V410L and L944I were not explicitly looked for in previous studies. The V410L mutation was nearing fixation in nearly all populations tested, with an overall allele frequency of 0.908, while the V1016G is found at 0.046 frequency. In contrast, the L944I mutation may have recently emerged, been introduced in the population, or is not under selection, as its allele frequencies remain low, ranging from 8.1% in Dorado to 17.9% in San Juan. As far as we are aware this is the first documentation of the L944I mutation in *Ae. aegypti* (L921I in *Ae. aegypti* numbering), although it may not have been investigated previously. However, it has previously been linked to pyrethroid resistance in other insect vectors and pests^28,29^. The role of the L944I mutation in pyrethroid resistance has been confirmed in *Drosophila melanogaster* by expressing the mutation in Xenopus oocytes. The conserved nature of the voltage-gated sodium channel and the mutation’s location in the critical region for pyrethroid binding in the *vgsc* domain II S4-S5 suggest that it may confer similar resistance phenotypes in *Aedes aegypti* (see alignment **SI** Fig. 2)^40^. The V410L and L944I mutations, not described in previous studies, highlight the advantages of the broader amplicon approach over traditional PCR methods. Additionally, we provide the first report of the *rdl* A296S mutation in the Puerto Rican *Ae. aegypti* population. This mutation, which confers resistance to organochlorines (cyclodienes and phenylpyrazoles), was found at an allele frequency ranging from 49.6 to 100%.

Linkage disequilibrium was observed among four non-synonymous mutations (V410L, V1016I, V1016G, and F1534C), with R² correlations ranging from 0.61 to 0.90. These mutations have demonstrated linkage in multiple *Ae. aegypti* populations^41–43^. Given the lack of regular or intensive vector control programs in Puerto Rico, the intensity of phenotypic resistance and the presence of genotypic markers are somewhat surprising. However, several previous studies have identified both phenotypic and genotypic resistance on the island. Additionally, ad hoc ultra-low volume (ULV) spraying of permethrin is conducted based on population demands or in response to Zika and dengue outbreaks. The recent outbreaks may explain the observed resistance and mutations, as increased implementation of control measures has likely exerted selective pressure on the mosquito population^44,45^. Alongside occasional spraying, the use of insecticides and pesticides in households and agriculture may have further contributed to the resistance profile in *Ae. aegypti* on the island. The combination of various active ingredients in these products, along with the anthropophilic nature of *Ae. aegypti*, likely leads to high exposure levels, promoting the development of the observed broad resistance profiles within the population.

The A296S mutation in the *rdl* gene, which encodes a GABA receptor chloride channel, was also detected in this population. This mutation is well-documented for its association with resistance to dieldrin, which was banned in 1970 due to concerns about its environmental impact and potential carcinogenic properties^46^. Despite this ban, the ongoing use of alternative insecticides or pesticides targeting the GABA chloride channel—such as cyclodiene organochlorines, phenylpyrazoles and pyrethroids (GABA is a secondary target) —may contribute to the persistence of this mutation^47,48^. The A296S mutation has been detected in various mosquito populations, including *Aedes aegypti* from Burkina Faso, Cape Verde, and Cameroon^16,27,49^, suggesting that it may not impose a significant fitness cost on these populations. However, conflicting evidence exists regarding this hypothesis^50,51^.

Our study revealed variations in resistance across different island locations when analysing phenotypic bioassay data. However, no differences were observed in the allele frequencies of insecticide resistance SNPs among these locations. This may suggest the limitations of bioassays in quantifying the intensity of insecticide resistance, or it could indicate a need for larger sample sizes to achieve more accurate quantification. We also highlight that, particularly in highly resistant populations, individual-level phenotype and genotype data is preferential for understanding phenotype-genotype associations and correlations. Alternatively, it may imply that other mechanisms contribute to the observed differences in insecticide resistance. Potential mechanisms include metabolic resistance or cuticular modifications, although these aspects were beyond the scope of our current study. Metabolic resistance has previously been documented in Puerto Rico through the use of piperonyl butoxide to isolate the effects of detoxifying enzymes^9^ and via RNA-seq^12^, which identified the overexpression of cytochrome P450 genes. Future research involving synergistic bioassay testing would be valuable for further investigating metabolic resistance in the identified locations. Additionally, future genotypic studies should focus on genes associated with metabolic resistance, such as P450 monooxygenases, esterases, and glutathione S-transferases. It is also crucial to examine copy number variants and genes linked to cuticular thickening, another recognized resistance mechanism, as highlighted by Faucon et al.^52^.

The WHO recognises the importance of molecular markers in understanding the evolving landscape of insecticide resistance mechanisms among medically significant vectors^7^. In our study, we employed a cost-effective and easily implementable assay that can screen numerous samples. By leveraging PCR multiplexing and dual barcoding, this approach enhances scalability and affordability, allowing for the pooling of amplicons across samples and facilitating discrimination during analysis. Additionally, these amplicons can be sequenced on various platforms, including portable sequencers like the Oxford Nanopore Technology MinION, making the method more applicable and accessible in low-resource settings. However, we acknowledge that sequencing technologies are not yet feasible for many vector control programs. Nevertheless, the Ebola and Zika outbreaks, along with the COVID-19 pandemic, have demonstrated the value of sequencing data for monitoring disease transmission. As a result, there has been increased investment in sequencing capabilities, leading to enhanced capacity in many countries.

Our work underscores the value of this methodology as a tool for identifying potential markers by revealing the presence of the novel L944I (L921I in *Aedes aegypti*) mutation. This mutation is associated with pyrethroid resistance in various species, including *Triatoma infestans* (the vector of *Trypanosoma cruzi*, the causative agent of Chagas disease in the Americas) and *Bemisia tabaci* (the silverleaf whitefly, a globally significant agricultural pest), highlighting the potential cross-species relevance of the identified genetic variation. While further studies are necessary to confirm the functional role of this mutation in *Aedes aegypti*, its homology across species emphasizes its potential utility in enhancing our understanding of insecticide resistance in both medically and agriculturally important arthropods^40,53^.

The complex landscape of phenotypic insecticide resistance in mosquitoes encompasses numerous contributing mechanisms and interactions. A deeper understanding of these mechanisms, particularly those related to metabolic resistance, could expand the genomic targets within the Amp-seq panel proposed in our study. This adaptable methodology can be utilised for surveillance of mosquito populations in conjunction with phenotypic testing, providing valuable insights to inform vector control programs that are essential for reducing disease burden.

## Materials and methods

### Sampling sites and bioassays

Mosquito eggs were collected from gravid ovitraps placed in five locations in Puerto Rico: Bayamón, Dorado, Guánica, Ponce, and San Juan (Fig. 1). Between April and May 2022, black cups containing seed germination paper were used as ovitraps, pre-prepared with hay infusion and deployed for one week at a time. The traps were placed within 50 m of residences. In compliance with the US Health Insurance Portability and Accountability Act of 1996 (HIPAA), we cannot disclose the exact locations of the traps. A total of 14 traps were deployed in Bayamón, 28 in Guánica, 27 in Ponce, and 36 in San Juan. For logistical reasons, only San Juan and Bayamón had multiple trap deployments, with three traps in San Juan and two in Bayamón. Between collections, the traps were washed. After one week of deployment, the traps were collected, and the oviposition papers were dried.

Eggs were reared in the Puerto Rico Vector Control Unit insectary according to standard laboratory protocols until they developed into adults. Adult mosquitoes, aged 3 to 5 days, were then tested for insecticide resistance using the CDC bottle bioassay with 250 mL Wheaton glass bottles. Technical deltamethrin was tested at 1 × (0.75 µg/bottle), 5×, and 10× doses, while technical malathion was tested at 1 × (400 µg/bottle), 2×, and 3× doses, following CONUS CDC recommendations^20^. An acetone control was included for both insecticides, and the ROCKEFELLER MR734 strain was used as a reference. The following reagent was obtained from BEI Resources, NIAID, NIH: *Ae. aegypti*, Strain ROCK, MRA-734, contributed by David W. Severson. After treatment, the uncapped bottles were placed on bottle rollers until completely dry.

Mosquitoes were morphologically identified as *Ae. aegypti* prior to testing by a trained entomologist using a key^54^. To test resistance, 18 to 25 F0 mosquitoes were placed in each bottle (3 treated with insecticide, 1 treated with acetone). Each experiment had a bottle for the ROCK susceptible control strain at diagnostic dose and a bottle of field caught mosquitoes exposed to acetone control. Knockdown of the mosquitoes was recorded every 15 min for 2 h as per CDC recommendations^20^. Mosquitoes were recorded as knocked down if they could no longer stand or fly. An assessment of resistance was made using the percentage knocked down at diagnostic time (30 min for deltamethrin and 15 min for malathion) for the diagnostic dose (0.75 µg/bottle for deltamethrin and 400 µg/bottle for malathion) as per CDC protocol^14,20^. Each bottle was used a maximum of two times before washing and recoating, control mortality showed this was acceptable for efficacy. Testing at each concentration and insecticide was repeated for as many times as number of mosquitoes collection allowed. Mosquitoes were preserved after phenotype testing in RNAlater^®^ and frozen at -80˚C.

### Molecular testing

Sample DNA was extracted using the Qiagen DNAeasy blood and tissue extraction kit following the manufacturer’s instructions. DNA concentration was tested using the Qubit 2.0 fluorophotometer. Subsequently, five-plex multiplex PCRs were carried out using Q5 High-fidelity PCR kits (New England Biolabs, UK), under the following conditions: initial denaturation (98.0 °C, 30 s) followed by 35 cycles of denaturation (98.0 °C, 10 s), annealing (57.3 °C, 35 s), and extension (72.0 °C, 45 s). 1 µL of DNA, 0.5 µL of each forward and reverse primer at 10 pmol/µL were combined with 19 µL of master to mix to make up to a 25 µL reaction (Tables S1, S2).

For Amp-seq, ten amplicons were designed (9 for insecticide resistance (4 genes), 1 for species identification). Amplicon primers were adapted from Collins et al., (2022), and changes were made to improve efficiency in multiplex combinations and target the *GSTe2* gene. Species primers were taken from Folmer et al.^22^, which target the cytochrome c oxidase subunit 1 (*cox-1*) gene of the mitochondria. Primer regions targeted single nucleotide polymorphisms (SNPs) within regions of ~ 500 base pairs (amplicon size). Index barcodes were eight base pairs in length (Tables S1, S2). A total of 17 SNPs across 4 genes; *vgsc*,* rdl*,* ace-1* and *GSTe2* were targeted with this panel (Table S3). PCR assays were carried out in the combinations outlined (Table S2), and the primers had 3’ barcodes attached to allow discrimination of individual samples; the barcodes used are outlined (Table S1). PCR products were visualised on 1% agarose gel with SYBR safe (Cambridge Biosciences, UK) alongside a 100 bp ladder. The products were purified with AMPure XP magnetic beads (Beckman Coulter), using a ratio of 0.8:1 (µL of beads to DNA). PCR assays were normalised to equal concentrations to create an overall pool of 20 ng/µL in 25 µL total volume (maximum of 10 amplicons across 50 barcoded mosquitoes = 500 amplicons). Sequencing was performed by Genewiz (Azenta Life Sciences) using Illumina MiSeq 250 bp pair-end reads, at a cost of ~£60 per pool or > US $0.15 per amplicon.

### Data and statistical analysis

Mosquito mortality levels were interpreted as per WHO/CDC criteria (98–100% - susceptibility, 90–97% - possible resistance, < 90% - resistance). Mortality is taken at the diagnostic time for the insecticide as per WHO guidelines. The mortality rate per site, insecticide and its concentration were modelled using a log-linear (Poisson) regression model with the (log) number of deaths as the outcome and an offset reflecting the (log) group sample size. Likelihood ratio tests were applied to determine the statistical significance of the covariates site, insecticide and concentration. Differences in allele frequencies across populations were estimated using Chi-squared tests and correlations between mortality and allele frequency assessed with Spearman’s rank test. All statistical analyses were performed using R (v4.3) software, with a significance level of 0.05.

### Bioinformatic analysis

A minimum of 50,000 reads were obtained per sample pool, and raw pooled FASTQ sequences were demultiplexed based on the 8 bp barcode primer-tag in each forward and reverse primer using an inhouse pipeline (available at https://github.com/LSHTMPathogenSeqLab/amplicon-seq). This pipeline removes and mis-tagging caused by errors in sequencing. Sequences were trimmed using *trimmomatic* software (v0.39) using the parameters LEADING:3, TRAILING:3, SLIDING WINDOW:4:20, MINLEN:36 to remove low quality ends of sequences^55^. Sequences were aligned to the *Ae. aegypti* reference (Vectorbase Aag2, GCA_021653915.1) using *bwa-mem* software and default parameters (v0.7.17-r1188). A small region of the intron in *vgsc* domain II amplicon did not map to the reference due to divergence in the sequences from Puerto Rico, therefore mapping was done to the *vgsc* domain II sequence for this amplicon (MK977835.1) (Fig. S3). Following mapping assessments of quality and mapping using *FastQC* (v0.12.1) and *samtools flagstat* (v1.17)^56^. Mapped reads were clipped using Samclip package. Variants were called using both *GATK* haplotype caller (v4.4.0.0, default parameters) and *freebayes* software (v1.3.6, default parameters), and filtered by *bcftools* to maximise confidence in the called SNPs^57,58^. Further filtering was carried out to ensure there was coverage across a minimum of 6 of the 10 amplicons.

Linkage disequilibrium was calculated using *vcftools* on phased vcf files created with *Beagle* (v 22Jul22.46e) software to provide a R^2^ value for pairwise combinations of non-synonymous mutations by sample country. Filtering was carried out based on the distance between mutations (minimum 20, maximum 10 Kbp). The related plots were generated using the *gaston* (v1.5.9) package in R.

Alignment of sequences to demonstrate the conservation of the L944I mutation across various species was performed in Aliview (v1.28) using the default MUSCLE alignment settings. The L944I mutation numbering is based on *Musca domestica* accession NW_026712250.

## Electronic supplementary material

Below is the link to the electronic supplementary material.

Supplementary Material 1

Supplementary Material 2

Supplementary Material 3

## Data Availability

Sequence data supporting the findings of this study have been deposited in the European Nucleotide Archive under project code PRJEB72548. Insecticide bioassay data and SNP VCF files are also included in the supplementary materials of this submission.
