# Supplementary material for: Profiling insecticide resistance phenotypes and genotypes in Aedes aegypti populations across four regions in Puerto Rico
Source: Sci Rep. 2025 Jul 18;15:26116. doi: 10.1038/s41598-025-03709-x (PMC12274374; doi:10.1038/s41598-025-03709-x)
Supplement: Supplementary file 1 — Supplementary Material 1 [file 41598_2025_3709_MOESM1_ESM.docx]

**SUPPLEMENTARY INFORMATION**

**S1 Table.** Barcodes used with primers sequences to distinguish samples

| **Direction** | **Name** | **Sequence** | **Direction** | **Name** | **Sequence** |
| --- | --- | --- | --- | --- | --- |
| Forward | BC1N | CTATCACG | Reverse | BC11N | ATGGCTAG |
| Forward | BC2N | TCCAGTGT | Reverse | BC12N | GACTTGGT |
| Forward | BC3N | GATCAGTA | Reverse | BC13N | TCGATCAC |
| Forward | BC4N | AGTGTCGG | Reverse | BC14N | ACACGTCA |
| Forward | BC5N | GTAGCGCT | Reverse | BC15N | CAATGTGC |
| Forward | BC6N | CATCTAAC | Reverse | BC16N | GGGACTAC |
| Forward | BC7N | TACAGATC | Reverse | BC17N | ACGTACTG |
| Forward | BC8N | CGTCTTGT | Reverse | BC18N | TGATTGCC |
| Forward | BC9N | TATGATCA | Reverse | BC19N | AACTCTAC |
| Forward | BC10N | GGTAGCTT | Reverse | BC20N | TGACTCAA |

**S2 Table. Amp-seq assay primers used in multiplex 1 and 2 combinations**

| **Multiplex 1** |  |  |  |
| --- | --- | --- | --- |
| **Forward Name** | **Forward** | **Reverse Name** | **Reverse** |
| DomainIV_F1 | GCGATCTSATCGAGAAGTA | DomainIV_R1 | ATGCTAGCAARTACGTGATG |
| DomainII_F5 | ACAATGTGGATCGCTTCCCG | DomainII_NR1 | TGAACCGAAATTGGACAAAAG |
| Ace_NF1 | TGGGGAACGCTRGGAATCTGCG | ACE_NR1 | GCATATCGCTGGGCAAACTC |
| LCO1490 | GGTCAACAAATCATAAAGATATTGG* | HCO2198 | TAAACTTCAGGGTGACCAAAAAATCA* |
| GSTE_111_F | ATGACGAAGCTCATTTTGTACACG | GSTE_111_R | ACAAAAATAAGCCACACACTACTG |
| **Multiplex 2** |  |  |  |
| **Forward Name** | **Forward** | **Reverse Name** | **Reverse** |
| DomainIIS4_F1 | TCTAGATTTAGYGACTCCAR | DomainIIS4_R1 | TACCGATGTAGTTCTTGCC |
| DomainI_F1 | TTTCGTCTAATGACCCAAGA | DomainI_R1_N | ARAGAWTTCGCTCACCCG |
| DomainIIIExon35_F2 | GGATCCAGATCATGAACGA | DomainIIIExon35_R1 | CATGTCGAACTTCTTATTGGT |
| RDL_NF1 | CTTCTAATTTCTCTCATCAC | Primer-Aeg-R1 | CTGGTTATTTGTACAAGTAGCA |
| GSTE_150_F | GAACCAATCCTTTTCGCC | GSTE_150_R | TGCCTTTTGAGCATTCTTCT |

*^22^

**S3 Table.** Average coverage per amplicon, amplicon length, and described mutations found in that amplicon.

| **Forward Amplicon Name** | **Reverse Amplicon Name** | **Mean Coverage** | **Amplicon Size** | **Insecticide Resistance mutations** |
| --- | --- | --- | --- | --- |
| Ace_NF1 | Ace_NR3 | 550.8 | 468 | G119S |
| DomainIIIExon35_F2 | DomainIIIExon35_R1 | 304.3 | 474 | T1520I, F1534C |
| DomainIIS4_F1 | DomainIIS4_R1 | 284.9 | 444 | G923V |
| DomainII_F5 | II_NR1 | 61.2 | 466 | L982W, S989P, I1011V/M, V1016I/G |
| DomainIV_F1 | DomainIV_R1 | 149.3 | 495 | D1763Y |
| DomainI_F1 | Domain1_R1_N | 244.5 | 464 | V410L |
| GSTE2_111 | GSTE2_111_R1 | 7.3 | 462 | L111S *(Ae. aegypti)*, C155F |
| GSTE2_150 | GSTE2_150_R | 256.0 | 321 | L119F, I150V *(Ae. aegypti)* |
| RDL_NF1 | Primer-Aeg-R1 | 40.4 | 482 | A301S |
| LCO1490 | HCO1298 | 6.5 | 709 | - |

**S4 Table. Summary of synonymous mutations detected in the 178 samples screened.** *Rdl* mutations were based on the AAEL008354-RF transcript, *vgsc* mutations utilised the AAEL023266-RL transcript and *ace-1* mutations refer to the AAEL034366-RD transcript.

| **Chrom** | **Gene** | **Position** | **Nucleic acid change** | **Annotation** | **Genotype** | | | **Alt. Allele Frequency** |
| --- | --- | --- | --- | --- | --- | --- | --- | --- |
|  |  |  |  |  | **Homo. ref** | **Hetero** | **Homo.**  **alt** |  |
| 3 | *ace-1* | 161500076 | T > A | 506T | 0 | 7 | 97 | 96.6% |
| 3 | *ace-1* | 161500136 | C > T | 486L | 102 | 4 | 0 | 1.9% |
| 3 | *ace-1* | 161500262 | G > A | 444D | 100 | 4 | 0 | 1.9% |
| 3 | *ace-1* | 161500301 | G > A | 431P | 100 | 4 | 0 | 1.9% |
| 3 | *ace-1* | 161500361 | T > C | 411I | 45 | 100 | 0 | 34.5% |
| 3 | *vgsc* | 315931749 | T > C | 1847Y | 16 | 91 | 0 | 42.5% |
| 3 | *vgsc* | 315932072 | G > A | 1762L | 179 | 1 | 0 | 0.3% |
| 3 | *vgsc* | 315932184 | G > A | 1724V | 88 | 2 | 0 | 1.1% |
| 3 | *vgsc* | 315932226 | G > C | 1710L | 80 | 80 | 0 | 25.0% |
| 3 | *vgsc* | 315984096 | C > T | 978L | 60 | 0 | 60 | 50.0% |
| 3 | *vgsc* | 315984129 | A > G | 967F | 0 | 0 | 62 | 100.0% |
| 3 | *vgsc* | 315984159 | C > T | 957K | 0 | 0 | 61 | 100.0% |
| 3 | *vgsc* | 316080738 | C > T | 402L | 225 | 10 | 1 | 2.5% |
| 3 | *vgsc* | 316081058 | G > A | 375D | 0 | 1 | 118 | 99.6% |

**S5 Table. SNPs nomenclature based on reference organism and *Ae. aegypti* from this study.**

| **Gene** | **Reference organism** | **Mutation reference** | **Mutation *Ae. aegypti*** | ***Ae. aegypti* transcript** |
| --- | --- | --- | --- | --- |
| *vgsc* | *Musca domestica* | V410L | V408L | AAEL023266-RL |
|  |  | L994I | L921I |  |
|  |  | V1016I/G | V1012I/G |  |
|  |  | F1534C | F1554C |  |
| *rdl* | *Drosophila melanogaster* | A296S | A301S | AAEL008354-RF |
| *Ace-1* | *Torpedo californica* | NA | NA | AAEL034366-RD |

**S6 Table.** Filtered linked SNP position with R^2^ > 0.8

| Position 1 | Position 2 | R^2^ | Position 1 | Position 2 | R^2^ |
| --- | --- | --- | --- | --- | --- |
| 161500136 | 161500301 | 1.000 | 315983956 | 315984096 | 0.943 |
| 315983762 | 316080895 | 1.000 | 315983973 | 315984096 | 0.943 |
| 315983762 | 316080896 | 1.000 | 315931749 | 315932226 | 0.934 |
| 315983762 | 316080903 | 1.000 | 315998798 | 316080620 | 0.911 |
| 315983762 | 316080916 | 1.000 | 316080722 | 316080983 | 0.909 |
| 315983809 | 315984129 | 1.000 | 315983762 | 315983763 | 0.899 |
| 315983956 | 315984130 | 1.000 | 315983763 | 316080895 | 0.899 |
| 315998609 | 315998611 | 1.000 | 315983763 | 316080896 | 0.899 |
| 315998609 | 315998782 | 1.000 | 315983763 | 316080903 | 0.899 |
| 315998610 | 315998613 | 1.000 | 315983763 | 316080916 | 0.899 |
| 316080830 | 316080845 | 1.000 | 316080830 | 316080847 | 0.895 |
| 316080895 | 316080896 | 1.000 | 316080845 | 316080847 | 0.895 |
| 316080895 | 316080903 | 1.000 | 316080952 | 316081058 | 0.857 |
| 316080895 | 316080916 | 1.000 | 315983762 | 316080942 | 0.856 |
| 316080896 | 316080903 | 1.000 | 315983762 | 316080950 | 0.856 |
| 316080896 | 316080916 | 1.000 | 316080895 | 316080942 | 0.856 |
| 316080903 | 316080916 | 1.000 | 316080895 | 316080950 | 0.856 |
| 316080942 | 316080950 | 1.000 | 316080896 | 316080942 | 0.856 |
| 315983809 | 315983956 | 0.986 | 316080896 | 316080950 | 0.856 |
| 315983809 | 315983973 | 0.986 | 316080903 | 316080942 | 0.856 |
| 315983809 | 315984159 | 0.986 | 316080903 | 316080950 | 0.856 |
| 315983956 | 315984129 | 0.986 | 316080916 | 316080942 | 0.856 |
| 315983973 | 315984129 | 0.986 | 316080916 | 316080950 | 0.856 |
| 315984129 | 315984159 | 0.986 | 315983763 | 316080830 | 0.850 |
| 315984096 | 315984159 | 0.972 | 315983763 | 316080845 | 0.850 |
| 315983956 | 315983973 | 0.971 | 315983762 | 316080847 | 0.844 |
| 315983956 | 315984159 | 0.971 | 316080847 | 316080895 | 0.844 |
| 315983973 | 315984159 | 0.971 | 316080847 | 316080896 | 0.844 |
| 315983809 | 315984096 | 0.957 | 316080847 | 316080903 | 0.844 |
| 315984096 | 315984129 | 0.957 | 316080847 | 316080916 | 0.844 |
| 316080722 | 316080942 | 0.954 | 315983762 | 316080722 | 0.816 |
| 316080722 | 316080950 | 0.954 | 315983762 | 316080983 | 0.816 |
| 316080942 | 316080983 | 0.954 | 316080722 | 316080895 | 0.816 |
| 316080950 | 316080983 | 0.954 | 316080722 | 316080896 | 0.816 |
| 315983762 | 316080830 | 0.945 | 316080722 | 316080903 | 0.816 |
| 315983762 | 316080845 | 0.945 | 316080722 | 316080916 | 0.816 |
| 316080830 | 316080895 | 0.945 | 316080895 | 316080983 | 0.816 |
| 316080830 | 316080896 | 0.945 | 316080896 | 316080983 | 0.816 |
| 316080830 | 316080903 | 0.945 | 316080903 | 316080983 | 0.816 |
| 316080830 | 316080916 | 0.945 | 316080916 | 316080983 | 0.816 |
| 316080845 | 316080895 | 0.945 | 316080830 | 316080942 | 0.808 |
| 316080845 | 316080896 | 0.945 | 316080830 | 316080950 | 0.808 |
| 316080845 | 316080903 | 0.945 | 316080845 | 316080942 | 0.808 |
| 316080845 | 316080916 | 0.945 | 316080845 | 316080950 | 0.808 |

**S1 Figure.** The log coverage of each amplicon sequenced separated by the multiplex reaction in which they were combined (see **Table 1**).


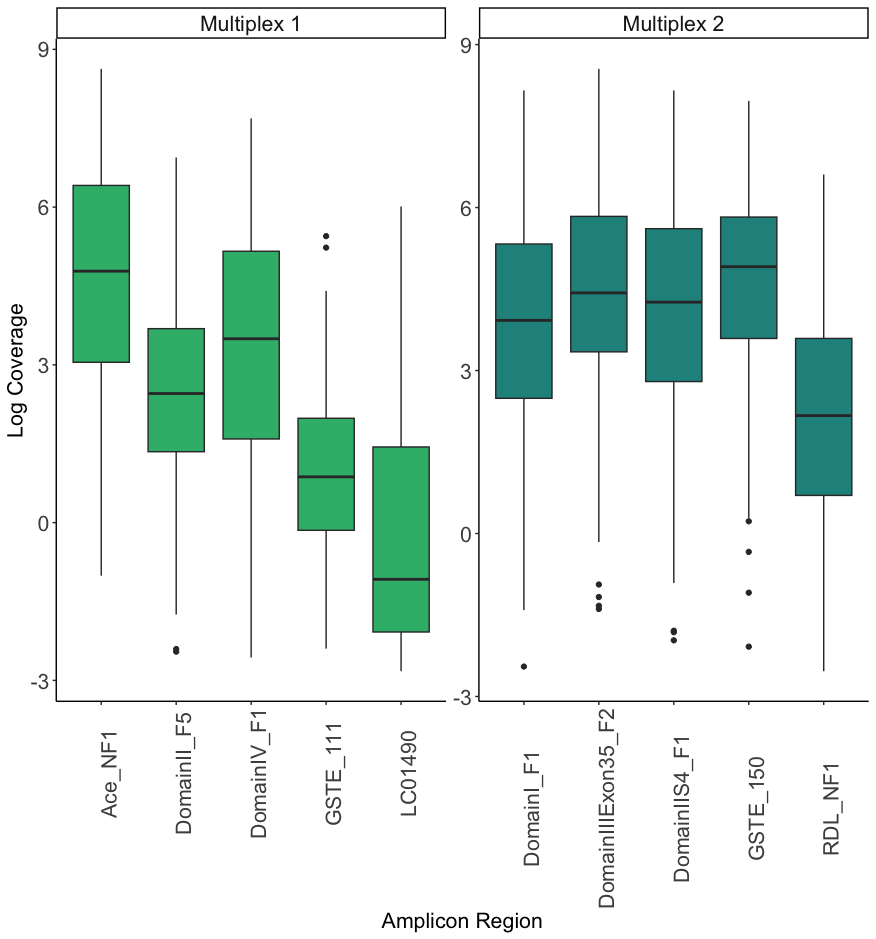


**SI Figure 2.** Alignment of protein for voltage-gated sodium channel for *Triatoma infestans* (sensitive AGW21773.1 and resistant AWG21772.1 forms), *Bemisia tabaci* and two isoforms of *Aedes aegypti* and *Drosophila melanogaster* showing position of L921I mutation described to be associated with pyrethroid insecticide resistance (28,29).

**
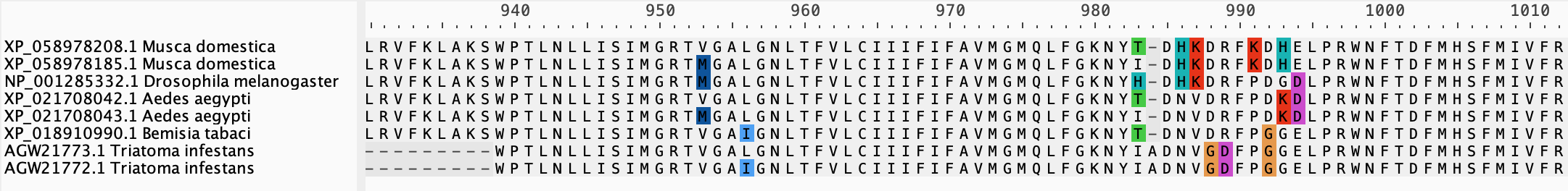
**
